# Carbapenemase-Producing *Klebsiella pneumoniae* in Romania: A Six-Month Survey

**DOI:** 10.1371/journal.pone.0143214

**Published:** 2015-11-23

**Authors:** Brandusa Elena Lixandru, Ani Ioana Cotar, Monica Straut, Codruta Romanita Usein, Dana Cristea, Simona Ciontea, Dorina Tatu-Chitoiu, Irina Codita, Alexandru Rafila, Maria Nica, Mariana Buzea, Anda Baicus, Mihaela Camelia Ghita, Irina Nistor, Cristina Tuchiluş, Marina Indreas, Felicia Antohe, Corinna Glasner, Hajo Grundmann, Aftab Jasir, Maria Damian

**Affiliations:** 1 Cantacuzino National Institute of Research-Development for Microbiology and Immunology, Splaiul Independentei 103, Bucharest, Romania; 2 The European Programme for Public Health Microbiology Training (EUPHEM), European Centre for Disease Prevention and Control (ECDC), Stockholm, Sweden; 3 Matei Bals National Institute of Infectious Diseases, Dr. Calistrat Grozovici Street 1, Bucharest, Romania; 4 Victor Babes Hospital for Infectious and Tropical Diseases, Mihai Bravu Road 281, Bucharest, Romania; 5 Elias Emergency University Hospital, Mărăsti Avenue 17, Bucharest, Romania; 6 Emergency University Hospital Bucharest, Splaiul Independentei 169, Bucharest, Romania; 7 Fundeni Clinical Institute, Fundeni Road 258, Bucharest, Romania; 8 Grigore Alexandrescu Emergency Children Hospital, Iancu de Hunedoara Avenue 30–32, Bucharest, Romania; 9 St. Spiridon Emergency Clinical Hospital, Piaţa Independenţei 1, Iasi, Romania; 10 Bacau County Emergency Hospital, Spiru Haret Street 2–4, Bacau, Romania; 11 Nicolae Simionescu Institute of Cellular Biology and Pathology, B.P. Hasdeu Street 8, Bucharest, Romania; 12 Department of Medical Microbiology, University Medical Center Groningen, Rijksuniversiteit Groningen Hanzeplein 1, 9713 GZ Groningen, Netherlands; Columbia University, UNITED STATES

## Abstract

This study presents the first characterization of carbapenem-non-susceptible *Klebsiella pneumoniae* isolates by means of a structured six-month survey performed in Romania as part of an Europe-wide investigation. *Klebsiella pneumoniae* clinical isolates from different anatomical sites were tested for antibiotic susceptibility by phenotypic methods and confirmed by PCR for the presence of four carbapenemase genes. Genome macrorestriction fingerprinting with *Xba*I was used to analyze the relatedness of carbapenemase-producing *Klebsiella pneumoniae* isolates collected from eight hospitals. Among 75 non-susceptible isolates, 65 were carbapenemase producers. The most frequently identified genotype was OXA-48 (n = 51 isolates), eight isolates were positive for *bla*_NDM-1_ gene, four had the *bla*_KPC-2_ gene, whereas two were positive for *bla*_VIM-1_. The analysis of PFGE profiles of OXA-48 and NDM-1 producing *K*. *pneumoniae* suggests inter-hospitals and regional transmission of epidemic clones. This study presents the first description of *K*. *pneumoniae* strains harbouring *bla*_KPC-2_ and *bla*_VIM-1_ genes in Romania. The results of this study highlight the urgent need for the strengthening of hospital infection control measures in Romania in order to curb the further spread of the antibiotic resistance.

## Introduction

Carbapenemase-producing Enterobacteriaceae (CPE), notably *Klebsiella pneumoniae*, produce serious infection (urinary tract infections, septicemia, pneumonia, and intra-abdominal infections) in debilitated and immunocompromised patients, in association with prolonged hospitalization and increased fatality ranging from 24% to 70%, depending on the study population [1]. CPE are spreading globally as multidrug-resistant pathogens for which there are only few treatment options available [1].

There are no national statistics in Romania regarding CPE or a national surveillance system for CPE, but a national sentinel surveillance system for nosocomial infections including invasive *Klebsiella pneumoniae* infections is operating. Also, as member of EARSNet, Romania is reporting yearly the number of invasive *K*. *pneumoniae* isolates which are resistant to carbapenems, but not the mechanisms of resistance, including carbapenemase producing capacity or type of enzyme. Clinical microbiology laboratories are currently performing the phenotypic diagnostic of carbapenem-non-susceptible isolates. Voluntary some of them which are investigating the carbapenemase-production capacity of *K*. *pneumoniae* isolates, are sending the strains for confirmation and further characterization to National Expert Laboratory (NEL) from Cantacuzino National Institute of Research-Development for Microbiology and Immunology (Cantacuzino NIRDMI).

In Romania, little is known about the distribution and spread of carbapenemase-producing *Klebsiella pneumoniae*, and the type of carbapenemases produced [2, 3, 4]. Only two studies have been published about CPE strains in Romania [3,4]. First study is describing the local distribution of carbapenemase encoding genes (*bla*_NDM-1_, *bla*_OXA-48_ and *bla*_OXA-181_) in nine clinical isolates collected in an emergency university hospital located in the center of Romania, whereas the second study presents the distribution of CPE in two hospitals in Bucharest during one year (2011–2012) [3, 4].

The present study was performed as part of the European Survey on Carbapenemase-Producing *Enterobacteriaceae* (EuSCAPE) [2] aiming to provide a six-months snapshot about the occurrence of the carbapenemase-producing *K*. *pneumoniae* isolated from clinical specimens of individual patients in Romanian hospitals. Moreover, this study identified the type of carbapenemase and determined the genomic diversity of carbapenemase-producing *K*. *pneumoniae* using Pulsed Field Gel Electrophoresis (PFGE). The results of this study are expected to contribute to a better understanding of the resistance genotype and geographical spread of carbapenemase-producing *K*. *pneumoniae* strains in Romania, which could inform more targeted infection control measures.

## Materials and Methods

### Study population

According to EuSCAPE protocol each participating country should enroll a number of hospitals in a geo-demographically representative manner, proportional with population size of the country. Romania is divided in eight developmental zones: north-western, north-eastern, western, center, southwest, southern, southern-east and Bucharest.

A number of twelve hospitals from every developmental zone of the country, having laboratory capacity to detect carbapenem resistance were invited in September 2013 to participate to the study. Six hospitals from Bucharest and two hospitals from Iasi and Bacau counties located in the north-eastern part of the country contributed with isolates. The population catchment of hospitals from capital city are comprising people living in Bucharest city but also in counties from southern and southern-east zones of Romania.

All hospitals participated on a voluntary basis to the project. During the six-month survey, starting in November 2013 ending in April 2014 (according to the EuSCAPE protocol), each hospital collected and dispatched the first 10 carbapenem-non-susceptible *K*. *pneumoniae* isolates from single successive patients to the NEL at the Cantacuzino NIRDMI for species confirmation, and further phenotypic and genotypic characterization. Each hospital also sent to NEL the information about the total number of *K*. *pneumoniae* isolates (both first successive non-susceptible and susceptible to carbapenem) enrolled in the study as well as the total number of isolates collected during the survey period (Table 1).

**Table 1:** Hospital origin, number and frequency of carbapenem-non-susceptible *K*. *pneumoniae* isolates included in the study and total number of carbapenem susceptible and non-susceptible isolates collected during survey period.

ID hospital number	Municipality/ county	Municipality/ county population size	Number of beds	Number of NS^a^ isolates sent to NRL for the study	Total number of isolates collected during survey period		
					NS (%)	S^b^ (%)	NS + S
H1	Bucharest	1 883 000	530	10	30 (16)	152 (84)	182
H2	Bucharest	1 883 000	265	5	5 (8)	57 (92)	62
H3	Bucharest	1 883 000	1084	10	42 (19)	178 (81)	220
H4	Bucharest	1 883 000	740	9	22 (21)	83 (79)	105
H5	Bucharest	1 883 000	441	7	12 (13)	80 (87)	92
H6	Bucharest	1 883 000	718	10	48 (18)	212 (82)	260
H7	Bacău	616 000	1182	12	52 (23)	172 (77)	224
H8	Iasi	772 300	1128	12	62 (26)	180 (74)	242

^a^carbapenem-non-susceptible isolates (NS)

^b^carbapenem susceptible isolates (S)

According to the protocol, the participating hospitals collected information about their bed size, catchment populations and some epidemiological data, such as age and gender of the patients, patient location within the hospitals (outpatient, normal ward, ICU), clinical relevance of the isolate (colonization versus infection), hospital acquisition or community onset, hospital admissions and history of travel during the previous six months.

### Bacterial strains

Bacterial isolates from hospitalized patients were identified in the hospital diagnostic laboratories using routine identification procedures [5], and antimicrobial susceptibility testing was performed by the disk diffusion method.

### Confirmation of carbapenem susceptibility

*K*. *pneumoniae* isolates were sent to the National Expert Laboratory and confirmed as carbapenem-non-susceptible by disk diffusion method using imipenem, meropenem and ertapenem disks. Screening for carbapenemase production was performed by a combination of Kirby-Bauer disk-diffusion methods according to the EUCAST guideline [6], MastDisc ID inhibitor combination disks (MDI) (Mast Group Ltd, UK) and the biochemical Carba NP II tests based on manufacturer’s instructions [7]. Moreover, a temocillin disk (30μg) was used for presumptive detection of OXA-48–like enzyme producers. MastDisc ID and Carba NP II were used for phenotypic confirmation of carbapenemase, whereas PCR assays were used for their genetic confirmation.

### Molecular detection and genetic characterization of carbapenemase encoding genes

Screening for the presence of most clinically relevant *K*. *pneumoniae* carbapenemases according to the EuSCAPE protocol was performed by amplification of the genes *bla*_OXA-48-like_, *bla*_NDM_, *bla*_KPC_ and *bla*_VIM_ using PCR primers described in Table 2.

**Table 2:** Primers used for screening, confirmation and sequencing of carbapenemase encoding genes.

Target gene	Primer	Sequence (5’-3’)	Amplicon size (pb)	Reference
Primers for screening				
*bla*_OXA-48-like_	OXA-F OXA-R	TTGGTGGCATCGATTATCGG GAGCACTTCTTTTGTGATGGC	744	[8]
*bla*_NDM_	NDM-F NDM-R	TGGCAGCACACTTCCTATC AGATTGCCGAGCGACTTG	488	[Miriagou, unpublished]
*bla*_KPC_	KPC-F KPC-R	CTGTCTTGTCTCTCATGGCC CCTCGCTGTRCTTGTCATCC	796	[9]
*bla*_VIM_	VIM-F VIM-R	AGTGGTGAGTATCCGACAG TCAATCTCCGCGAGAAG	212	[10]
Primers for confirmation and sequencing				
*bla*_NDM_	NDM-Fseq NDM-Rseq	GCAGCTTGTCGGCCATGCGGGC GGTCGCGAAGCTGAGCACCGCAT	782	[11]
*bla*_KPC_	KPC-Fseq KPC Rseq	ATGTCACTGTATCGCCGTCT TTTTCAGAGCCTTACTGCCC	883	[12]
*bla*_VIM_	VIM-Fseq VIM-Rseq	GTTTGGTCGCATATCGCAAC AATGCGCAGCACCAGGATAG	801	[13]

Total genomic DNA from 75 carbapenem-non-susceptible *K*. *pneumoniae* strains used in all PCR assays was obtained by thermal lysis (boiling a fresh cultured colony in 200 μl distilled water and subsequent centrifugation at 14,000 rpm).

For further characterization of genes encoding NDM, VIM and KPC carbapenemases a second PCR was performed with specific primers (Table 2). For OXA-48-like carbapenemase the same primers used for PCR screening were used for sequencing. Obtained amplified products were sequenced using BigDye Terminator v 3.1 and 3100 Avant Genetic Analyser (Applied Biosystems). The sequences were edited and aligned using the BioEdit version 7.0.5.3. software package.

The genetic relatedness among carbapenem-non-susceptible isolates was evaluated using PFGE analysis according to the standard protocol designed by the Centre for Disease Control and Prevention Atlanta (http://www.cdc.gov/pulsenet/pathogens/index.html), and recommended within the ECDC study project for molecular typing of bacteria involved in food and water diseases (FWD) [14]. PFGE of *Xba*I-digested total DNA was performed as described for *E*. *coli*. PFGE profiles were compared using BioNumerics v 5.1. We defined a PFGE type based on a similarity cut-off of 80% (Dice coefficient, represented by UPGMA, 1.5% optimization and 1.5% tolerance). Different PFGE types were given in alphabetical order. Every band difference within a PFGE type resulted in a subtype which was given a numerical order.

### Ethics Statement

Informed written consent was obtained from all participants in this study after explanation of the procedure and the purpose of the study. The study was approved by the review boards of the Research Ethics Committee, Cantacuzino National Institute of Research-Development for Microbiology and Immunology.

## Results

### Bacterial isolates

All the 75 isolates preliminarily identified in the hospital laboratories participating in the EuSCAPE project were confirmed as *K*. *pneumoniae* by the National Expertise Laboratory in Cantacuzino NIRDMI. They originated from different anatomical sites: urine (n = 39), lower respiratory tract (n = 17), blood (n = 13), wound (n = 4), puncture sites (n = 1) and peritoneum (n = 1).

About 51% of carbapenemase-producing isolates were from patients with hospital-acquired infections, 26% had a community onset, whereas for 23% the epidemiological context was unknown. The history of previous hospitalization and travel remained unknown for all patients.

### Phenotypic characterization of carbapenem-non-susceptibility

The results of the Kirby-Bauer disk-diffusion method showed that 72 (96%) of *K*. *pneumoniae* isolates were resistant to ertapenem, 40 (50.3%) exhibited meropenem resistance, whereas only 8 (10.7%) of the isolates were imipenem resistant.

Phenotypic tests for carbapenemase detection showed that 65 out of 75 carbapenem-non-susceptible isolates contained one of three Ambler classes of carbapenemase, A (KPC-type), B (metallo-beta-lactamase—MBL) or D (OXA-type), whereas for 10 isolates no carbapenemase activity was detected.

For 51 isolates, testing for temocillin susceptibility showed absence of inhibition zones in the disk diffusion test (disk load 30μg) indicative of OXA-48-like carbapenemase. MastDisc ID and Carba NP II identified 10 isolates positive for class B carbapenemase, 4 isolates as class A carbapenemase producers, whereas the remaining 51 carbapenemase-producing isolates could be suspected as class D carbapenemase producers.

### Genetic confirmation of carbapenemase-producing isolates

#### Distribution of genes encoding carbapenemases among carbapenemase-producing isolates

The PCR results showed that 51 strains harbored the *bla*_OXA-48-like_ gene, 8 strains had the *bla*_NDM_ gene, whereas the *bla*_KPC_ and *bla*_VIM_ genes were present in 4 and 2 strains, respectively.

*bla*_OXA-48-like_ gene positive *K*. *pneumoniae* isolates were identified in all hospitals, while *bla*_NDM_ was detected in half and *bla*_KPC_ and *bla*_VIM_ were identified in only three and one of the participating hospitals, respectively (Table 3). Among the remaining 10 carbapenem-non-susceptible isolates none of the four carbapenemase target genes were detected.

**Table 3:** Distribution of carbapenemase-producing isolates according to hospital of isolation.

Hospital	Number carbapenems- non-susceptible	Number carbapenemase producers	Type of carbapenemase			
			OXA-48	NDM	KPC	VIM
H1	10	8	6	2	-	-
H2	5	5	4	1	-	-
H3	10	8	4	2	2	-
H4	9	8	7	-	1	-
H5	7	6	5	-	1	-
H6	10	9	9	-	-	-
H7	12	10	8	-	-	2
H8	12	11	8	3	-	-
Total	75	65	51	8	4	2

#### The sub-type of carbapenemase genes among carbapenemase-producing isolates

DNA sequencing allowed for an identification of the sub-type of the respective carbapenemase genes in all carbapenemase-producing *K*. *pneumoniae* isolates. Thus, all sequenced KPC producers’ harboured *bla*_KPC-2_ genes, whereas all OXA-48-like producers had a *bla*_OXA-48_ gene. All NDM producers had a *bla*_NDM-1_ gene, whereas all VIM producers carried the *bla*_VIM-1_ gene.

#### The genetic relatedness among carbapenemase-producing isolates

**Fig 1:**
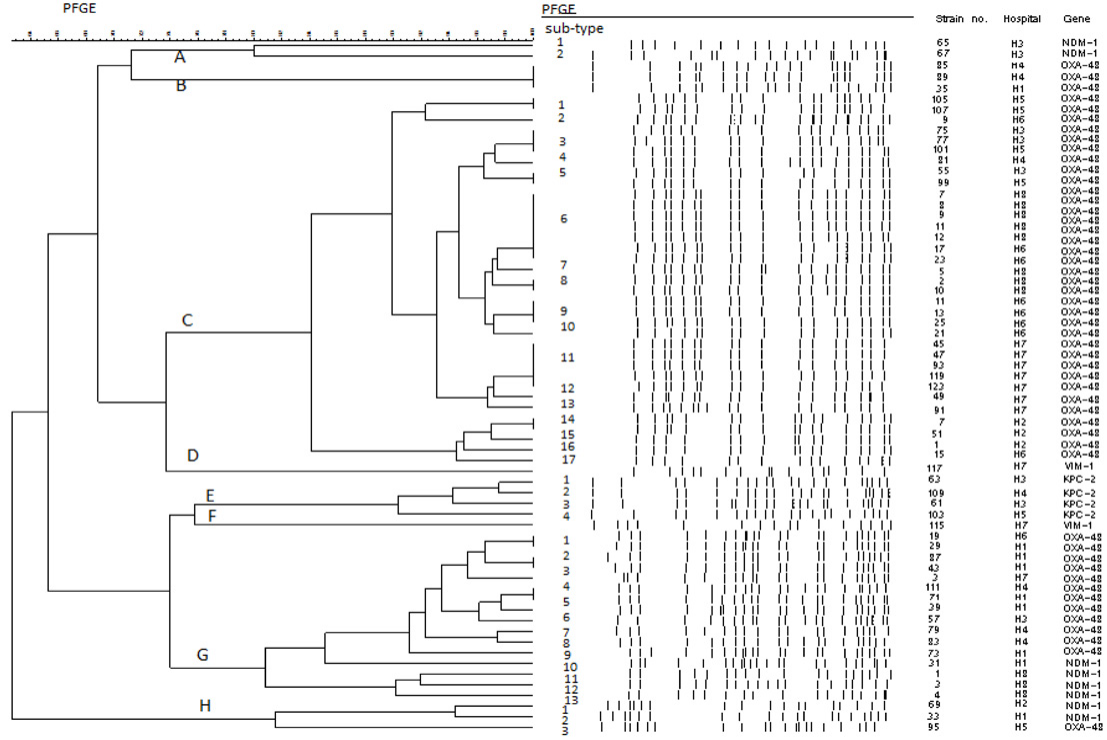
PFGE profiles of 65 carbapenemase-producing *Klebsiella pneumoniae* isolates.

The analysis of PFGE results of the 65 carbapenemase-producing *K*. *pneumoniae* isolates identified 8 PFGE types (i.e. A-H) (Fig 1). The most frequent type was C, which consisted of 35 isolates subdivided into 17 PFGE subtypes (i.e. C1-C17) followed by G with 16 isolates with 13 subtypes (i.e. G1-G13) (Fig 1). Most OXA-48 producers, 47 (92%) isolates had these genotypes. The minor types (i.e. A, B, D, E, F, and H) included between 1 and 4 isolates each. Among them, type E was exclusively represented by KPC-2-positive isolates.

## Discussion

This is the first systematic multi-centre study describing the occurrence of carbapenem-non-susceptible *K*. *pneumoniae* isolates and their genetic resistance determinants responsible for carbapenemase production in Romania.

Our study shows that OXA-48 carbapenemase has been the most prevalent carbapenemase during the study period, being detected in 79% of carbapenemase-producing *K*. *pneumoniae* isolates in Romanian hospitals. Importantly, strains that produce this carbapenemase were spreading among all participating hospitals in this study. OXA-48 had first been identified from a clinical *K*. *pneumoniae* isolate recovered in Istanbul, Turkey, in 2001 [15]. For several years, OXA-48 appeared to be confined to Turkey, as almost all OXA-48 beta-lactamase producers were reported either among patients hospitalized in Turkey or with a link to that country [16, 17]. Since 2008, this carbapenemase has been identified in many other countries, mainly in *K*. *pneumoniae* [15, 17]. In addition to sporadic cases, an increasing number of outbreaks due to OXA-48-producing *K*. *pneumoniae* are currently reported, not only in Turkey but also in Belgium, France, Greece, the Netherlands and Spain [15, 17–20]. The successful spread of OXA-48 producers is now considered an epidemic threat as it represents an important source of multidrug resistance in *K*. *pneumoniae* in Europe [21, 22].

In Romania the presence of OXA-48 producing *K*. *pneumoniae* isolates has only occasionally been reported. The first study published was conducted between January 2010 and September 2012 in an emergency university hospital from the central part of Romania, where three *K*. *pneumoniae* ST101 isolates with indistinguishable PFGE profiles were reported [3]. The second study was performed between October 2011 and November 2012 in two hospitals in Bucharest, which identified 31 patients with *K*. *pneumoniae* isolates harbouring *bla*_OXA-48_ gene [4]. No typing results were reported.

In the present study PFGE analysis of the 51 OXA-48 producing *K*. *pneumoniae* strains showed that 47 out of 51 (92%) strains isolated from all hospitals belonged to the same epidemic clone, whereas the remaining four isolates had different PFGE profiles. These results suggest an epidemic expansion of a major OXA-48 positive clone which evolves hospitals in different regions of the country.

We also identified eight *K*. *pneumoniae* isolates harbouring the *bla*_NDM-1_ gene. This gene may be carried by different plasmid types, most of them co-harboring multiple but variable resistance determinants [23]. A few plasmids carrying *bla*_NDM-1_ also encode other carbapenemases, including OXA-181- and VIM-types [24]. The first New Delhi metallo-beta-lactamase (NDM-1) producing strains detected in Europe were associated with importation from the Indian subcontinent. More recently, countries in the Balkan region (Serbia, Montenegro, Bosnia and Herzegovina) also reported patients infected and colonized with NDM-1 producers [23, 25–26]. In Romania, the presence of this enzyme among *K*. *pneumoniae* was recognized previously [3, 4]. In addition, anecdotal reports suggested the introduction of NDM-1 producing strains by travelers into Romania [27]. Their PFGE profiles exhibited a ≥ 80% similarity, indicative of a single clone, expanding within and between four hospitals from different Romanian regions. According to an international staging system [28] Romania thus would assume an epidemiological stage 4 for both OXA-48 and NDM-1 producing *K*. *pneumoniae*.

We also found isolates producing VIM-1 as well as KPC-2 carbapenemase. Strains with these types had previously not been identified in Romania. The PFGE profiles of two VIM-1 positive strains collected from patients hospitalized in the same hospital (H7) were seemingly different, showing a similarity < 80%. The PFGE profiles of four *K*. *pneumoniae* isolates harbouring *bla*_KPC-2_ gene were however rather similar, with similarity ranging from 90% to 98%, being part of the epidemic clone, showing both intra- and inter-hospitals (H3, H4 and H5 hospitals) transmission.

The PFGE results of this study are testimony of a clonal dissemination of *K*. *pneumoniae* harbouring all of the four carbapenemase genes across the sample of Romanian hospitals which have been enrolled and bears the hallmark of a much larger i.e. national epidemic of carbapenemase-producing *Enterobacteriaceae* in Romanian hospitals.

Hospitalized patients from whom the carbapenemase-producing *K*. *pneumoniae* isolates were collected where kept in contact isolation according to guideline regarding prevention of hospital infection transmission.

The results of PCR tests performed on 10 carbapenem resistant strains with no carbapenemase type detected by phenotypic tests showed that in these strains none of the carbapenemase targeted genes were detected, suggesting that other resistance mechanisms to carbapenems (ESBL_S_ or AmpC β-lactamases combined with the loss of outer membrane porins OmpK35 and/or OmpK36) could have been involved [29, 30]. It could also be possible that carbapenemases other than those included in this study may have caused resistance in these isolates. But this seems unlikely given the negative results of our phenotypic confirmation tests and the scarcity with which other carbapenemases are reported in Europe. These results demonstrate the importance of molecular tests in the confirmation of carbapenemase-producing *Klebsiella pneumoniae*.

### Limitations of the study

This study has several limitations regarding the rate of response of 67% of invited hospitals participating in the survey as well as the time of only six months. Although hospitals were invited in a geo-demographically representative manner the selection bias could occur due to the fact that participating hospitals in the survey were those having a catchment population for counties from southern, southern-east, north-eastern and Bucharest zones of Romania and obtained data could be extrapolated only for those zones. Clinical data of the isolates as well as data on antibiotic consumption are not presented in this study.

## Conclusions

This study shows the first detection of *K*. *pneumoniae* strains harbouring *bla*_KPC-2_ and *bla*_VIM-1_ genes in Romania. The carbapenemase most frequently detected is OXA-48, representing 79% of carbapenemase-producing *K*. *pneumoniae* strains. This carbapenemase was detected in isolates collected from patients hospitalized in all of the participating hospitals, and the PFGE results suggest the spread of a single clone in all sampled hospitals with outbreaks in two of them. Likewise, NDM-1 positive strains have also been identified in four hospitals and PFGE results indicate a similar epidemic dynamic as for OXA-48. Based on these findings Romania would assume a epidemiological stage 4 for OXA-48 and NDM-1 carbapenemases according to an international staging system.

Before starting this survey by self-assessment realized by the national expert in March 2013, the epidemiological stage 1 was established for carbapenemase-producing isolates from Romania. The present study allowed updating and upgrading the epidemiological stages for all investigated carbapenemases.

The results of this study show that for efficient monitoring of the rapidly evolving spread of carbapenemase-producing *K*. *pneumoniae* the development of a national management system for CPE through a dedicated surveillance program, the provision of references services, the obligation to notify cases to health authorities and a national plan for containment of/or preparedness to contain CPE are necessary. In addition European Council Recommendation of 15 November 2001 [31] on the prudent use of antimicrobial agents in human medicine should strongly be considered including Strategies towards sufficient surveillance systems on usage of antibiotics and antimicrobial resistance, control and preventive measures, promoting education and training of health care workforce and inform the general public.
